# Subjective spatial orientation discomfort is associated with decreased real-world spatial performance and lower cognitive function

**DOI:** 10.3389/fnins.2024.1481653

**Published:** 2024-11-13

**Authors:** Johannes Gerb, Vivien Oertle, Sandra Becker-Bense, Thomas Brandt, Marianne Dieterich

**Affiliations:** ^1^German Center for Vertigo and Balance Disorders, LMU University Hospital, Munich, Germany; ^2^Graduate School of Systemic Neuroscience, LMU Munich, Munich, Germany; ^3^Hertie Senior Professor for Clinical Neuroscience, LMU Munich, Munich, Germany; ^4^Department of Neurology, LMU University Hospital, Munich, Germany; ^5^Munich Cluster for Systems Neurology (SyNergy), Munich, Germany

**Keywords:** visuospatial, spatial anxiety, spatial discomfort, dementia, cognitive impairment, pointing task, egocentric, allocentric

## Abstract

**Background:**

Spatial memory and orientation deficits often precede cognitive impairment in incipient dementia, e.g., Alzheimer’s disease. Therefore, early diagnosis of spatial impairment may be crucial to the initiation of an adequate therapeutic intervention. Subjective tests, such as spatial anxiety and spatial discomfort questionnaires, and objective tests in the form of quantitative measures of orientation, are available. In these tests, vestibular hypofunction has often been neglected as a potential confounder. The major research question in this study was how self-assessed questionnaires correlate with the data from objective measures in participants with proven normal vestibular function.

**Methods:**

A heterogeneous group of 135 participants (72 females, 63 males, mean age 62.75 ± 14.46 years) from a tertiary center for vertigo and balance disorders consisting of two cohorts, with (*n* = 49) and without (*n* = 86) cognitive deficits in a screening test (MoCA), was examined (a) with a newly introduced inventory for subjective spatial discomfort (Extended Inventory for Spatial Orientation Discomfort, EISOD), (b) a well-established questionnaire for subjective spatial skills (Santa Barbara Sense of Direction Scale, SBSODS), and (c) the objective three-dimensional real-world pointing task (3D-RWPT) before and after horizontal body rotations. In all patients, acute central or peripheral vestibular deficits were ruled out by neuro-orthoptics, bithermal water calorics and video head impulse testing.

**Results:**

Self-assessed spatial orientation discomfort (EISOD) correlated with the amount of spatial impairment in the 3D-RWPT for both cohorts. The cognitively impaired patients showed significantly higher levels of spatial discomfort (i.e., lower scores; Welch’s *t*-test t-2.58, *p* < 0.01, Cohen’s d − 0.46), and higher angular deviations in the (cognitively demanding) transformation paradigm of the 3D-RWPT (t 2.37, p 0.02, Cohen’s d 0.44). They preferred retinotopic/egocentric spatial encoding strategies in the pointing task (Welch’s *t*-test t-2.61, *p* < 0.01, Cohen’s d − 0.47). In contrast, the self-report of spatial abilities (SBSODS) yielded no significant group differences (t − 1.66, p 0.10) and was not reliably associated with objective accuracy in the pointing task.

**Conclusion:**

In patients without vestibular deficits, subjective spatial discomfort (EISOD) correlated with the accuracy in an objective 3D-pointing task for both cohorts, and higher discomfort was associated with more severe cognitive impairment. EISOD-scores showed higher correlation indices than a self-report of spatial skills using the SBSODS. When investigating spatial abilities in patients with suspected cognitive impairment, it appears reasonable that both subjective spatial discomfort, subjective spatial abilities, and objective spatial measures should be combined. Future research in patients with vestibular dysfunction is needed to understand the role of vestibular deficits for the development of spatial orientation discomfort.

## Introduction

Visuospatial orientation deficits commonly occur in early stages of Alzheimer’s disease (AD) (Plaza-Rosales et al., 2023) and other forms of dementia (Čepukaitytė et al., 2024). Similar to neuroimaging parameters, which can be observed years before clinical dementia onset (Gordon et al., 2018), spatial impairment often precedes the cognitive decline (Allison et al., 2016). Early diagnosis of spatial impairment may be crucial to allow for early intervention. Therefore, multiple molecular, psychophysical and behavioral biomarkers have been investigated (Tangen et al., 2022; Puthusseryppady et al., 2022). Further, it is important to know that sensory deficits such as chronic bilateral vestibulopathy (BVP) can also cause significant spatial impairment, associated with hippocampal atrophy (Smith, 2022; Brandt et al., 2005; Kremmyda et al., 2016; Zwergal et al., 2023). For an adequate personalized treatment, disorders of cognitive impairment and peripheral vestibular loss, which may occur as separate or combined conditions, need to be differentiated (Gerb et al., 2024a; Bosmans et al., 2022; Obermann et al., 2023; Wei et al., 2018).

A number of tests measuring objective spatial abilities are available, such as neuropsychological orientation tasks, digital navigation in virtual environments, performance of real-world orientation and navigation, or tasks with a stationary subject-world interaction [for a recent overview and detailed analysis of practical and conceptual pitfalls, see (Uttal et al., 2024)]. A recently established three dimensional real-world pointing task (3D-RWPT) (Gerb et al., 2023a) provides a simple and fast measure for the differentiation of spatial memory deficits in patients with dementia or bilateral vestibular failure as a bedside test (Gerb et al., 2024a). In short, the 3D-RWPT requires the sitting participant to update their reference frame of the environment after horizontal whole-body rotations (i.e., following a yaw-axis rotation) in order to still be able to correctly interact with static real-world targets (Gerb et al., 2024a).

In contrast to the above-described objective 3D measurements of spatial orientation, subjective questionnaires are available based on the self-assessments that require individuals to estimate their spatial abilities and indicate their anxiety/discomfort level in various hypothetical spatial orientation tasks. However, a patient’s self-estimated navigation ability is influenced by several factors leading to subjective misjudgments (Weisberg et al., 2014; Carbone et al., 2019; Taillade et al., 2015), and cannot reliably predict vestibular deficits (Moore et al., 2024; Gerb et al., 2024b). Spatial anxiety or spatial orientation discomfort is a common finding in mild cognitive impairment (MCI) and AD (Cerman et al., 2018). Importantly, older test instruments for spatial anxiety, e.g., by Lawton (1994), focus on participant discomfort during imagined navigation tasks, i.e., only one aspect of spatial abilities. Other relevant domains of spatial abilities, such as mental rotation or mental imagery (Shah and Miyake, 2012), were not part of the test.

In order to develop a balanced, easily applicable test instrument for spatial orientation discomfort / anxiety, we modified and extended previously validated spatial anxiety questionnaires and spatial anxiety scales (Lawton, 1994; Lyons et al., 2018; Geer, 2019). This test instrument was then used in a heterogeneous patient group of two cohorts, with either normal cognition or mild to moderate cognitive impairment. All patients participated in the 3D-RWPT, allowing for correlation analyses between cognitive test scores, 3D-RWPT performance, a self-assessment of spatial abilities, and the novel test instrument for spatial orientation discomfort. All patients enrolled had normal vestibular and ocular motor function. It should be noted that the final diagnosis or phenotypical investigation of distinct neuropathological subtypes of cognitive impairment were not aim of this methodical work.

## Methods

### Construction of a spatial discomfort scale with various subdomains

Previously validated test items from Lyons et al. (2018), Lawton (1994), and the work by Geer (2019) led us to compile a 16-item questionnaire for the fast assessment of various aspects of spatial anxiety and discomfort. The so-called extended Inventory for Spatial Orientation Discomfort (EISOD) consists of four questions on hypothetical mental imagery (MI) tasks, four questions on mental manipulation (MM) scenarios, four questions involving navigation (N) and four questions involving scalar abilities (SA). For all questions, participants should provide their hypothetical stress / anxiety level on a five-point Likert scale (ranging from 1: very anxious / highly stressed to 5: no anxiety / no stress). The resulting scores range from 1.0 (severe spatial anxiety in all hypothetical scenarios) to 5.0 (no relevant spatial anxiety in any of the hypothetical scenarios) and can be calculated for the respective subdomains (MI, MM, N, SA) individually. The test instrument can be found in the Supplementary data.

### Patients

The patients enrolled in the current study were examined in our tertiary interdisciplinary German Center for Vertigo and Balance Disorders and the Department of Neurology, Ludwig-Maximilians-University, Munich, Germany, between 10/2023 and 06/2024. Patients with acute or chronic uni- or bilateral vestibulopathy, relevant impairments of arm function due to, e.g., paresis, ataxia, tremor, or orthopaedic disorders, as well as patients with uncorrected visual or hearing loss were excluded from the study. The patients enrolled were presenting due to various neurotological and non-neurotological reasons, for instance, after an episode of probable benign paroxysmal positional vertigo [BPPV (von Brevern et al., 2015)], in interictal intervals of suspected vestibular migraine (VM) (Lempert et al., 2022), with gait instability due to polyneuropathy, unsteadiness due to orthostatic dysregulation, or due to functional dizziness (Staab et al., 2017). One of the authors (JG) decided on patients’ eligibility for inclusion based on the current clinical findings and patients’ records, ensuring that only patients with normal vestibular function were included. Additionally, patients with definite VM or acute BPPV were excluded from the analysis. To rule out potential confounders related to non-neurotological disorders, additional subgroups were created for polyneuropathy (PNP; normal sensory input vs. PNP) and main diagnosis time course (episodic vs. chronic symptoms). The patient cohort did not overlap with those previously published by our group (Gerb et al., 2024a; Gerb et al., 2024b; Gerb et al., 2023b).

The data protection clearance and Institutional Review Board of the Ludwig-Maximilians-University, Munich, Germany, approved the study (no. 094–10) and all patients gave informed consent. The study was performed in accordance with the ethical standards laid down in the 1964 Declaration of Helsinki and its later amendments.

### Clinical and neurotological testing

Clinical testing included a neurological and neuro-orthoptic examination, i.e., spontaneous and head-shaking nystagmus, ocular motor examination, fundus photography and adjustment of the subjective visual vertical [SVV, in order to detect central vestibular deficits and acute vestibular tonus imbalances (Dieterich and Brandt, 1993; Brandt and Dieterich, 2017)], bithermal water caloric testing [to measure the function of the horizontal semicircular canals in the low-frequency range of the vestibulo-ocular reflex (Jongkees et al., 1962)] and standardized video-head-impulse-test measurements of the semicircular function in the high-frequency range (Halmagyi et al., 2017) using the EyeSeeCamHIT^®^ system (EyeSeeTec, Munich, Germany) (Brandt et al., 2022).

### Psychometric testing

The psychometric questionnaire battery consisted of (i) the Santa Barbara Sense of Direction Scale (SBSODS) (Hegarty, 2002), (ii) the Patient Health Questionnaire subsection 9 (PHQ-9) (Kroenke et al., 2001), (iii) the Edinburgh Handedness Inventory (EHI) (Oldfield, 1971), (iv) the Montreal Cognitive Assessment (MoCA) (Nasreddine et al., 2005), (v) the aforementioned, newly-compiled extended inventory for Spatial Orientation Discomfort (EISOD), and (vi) the German version of the state/trait anxiety inventory (Spielberger et al., 1971) in its short version. Complete psychometric testing took 15–20 min depending on patients’ compliance and cognitive level. SBSODS, PHQ-9, EHI and state/trait anxiety inventory were filled out by the participants themselves without supervision or time constraints, while the MoCA screening test was performed in a standardized fashion by a medical doctor or a trained doctoral student. Suspected cognitive impairment was defined by scores lower than 26 points in the MoCA after correction for patient education level (Kessels et al., 2022).

The SBSODS (Hegarty, 2002) is a commonly applied psychometric test instrument for subjective spatial abilities. It consists of 15 questions on different aspects of spatial abilities, which the participants answer on a 7-point Likert scale, resulting in a mean score of 1.0–7.0 (with lower scores demonstrating worse self-assessed spatial skills). We analyzed the SBSODS self-report as a whole and subdivided into three item groups: items with an emotional element (e.g., “I do not enjoy giving directions.”), items centered on absolute self-assessed function (e.g., “I am very good at judging distances.”), and items where a higher score does not necessarily indicate a better spatial performance but possibly individual preferences (e.g., “I tend to think of my environment in terms of cardinal directions (N, S, E, W).”). Each item was classified in the same way as described in prior studies [emotional subset: items 6, 7, 8, 10, 13; functional subset: items 1, 2, 3, 4, 9, 10, 14; neutral subset: items 5, 12, 15 (Gerb et al., 2024a)]. For the EISOD, both overall scores and the four subsets (MI, MM, N, SA) were analyzed.

The Patient Health Questionnaire subsection 9 (PHQ-9) (Kroenke et al., 2001) consists of nine questions about the occurrence of depressive symptoms in the past two weeks with semi-quantitative answer options. The resulting score (0–27 points) can be used as a depression screening tool, with scores <10 being considered normal, and higher scores being suggestive of depression.

The Edinburgh Handedness Inventory (EHI) (Oldfield, 1971) aims to objectively ascertain the handedness of a subject. It consists of ten activities of daily living, for which the subject is asked to specify which hand (left or right) they prefer to perform said activity. Based on the answers, a laterality quotient can be calculated. This questionnaire was used to determine which patient hand to use in the 3D-RWPT.

The state/trait anxiety inventory (Spielberger et al., 1971) is a commonly used inventory aiming to measure different components of anxiety. The short version consists of two sets of ten questions on current emotional state (state anxiety) and general emotional state (trait anxiety) which are answered on an 8-Point Likert scale. The mean score can be analysed as raw values (10–80 points) or as a percentage (0–100%).

### 3D real-world pointing task (3D-RWPT)

**Figure 1:**
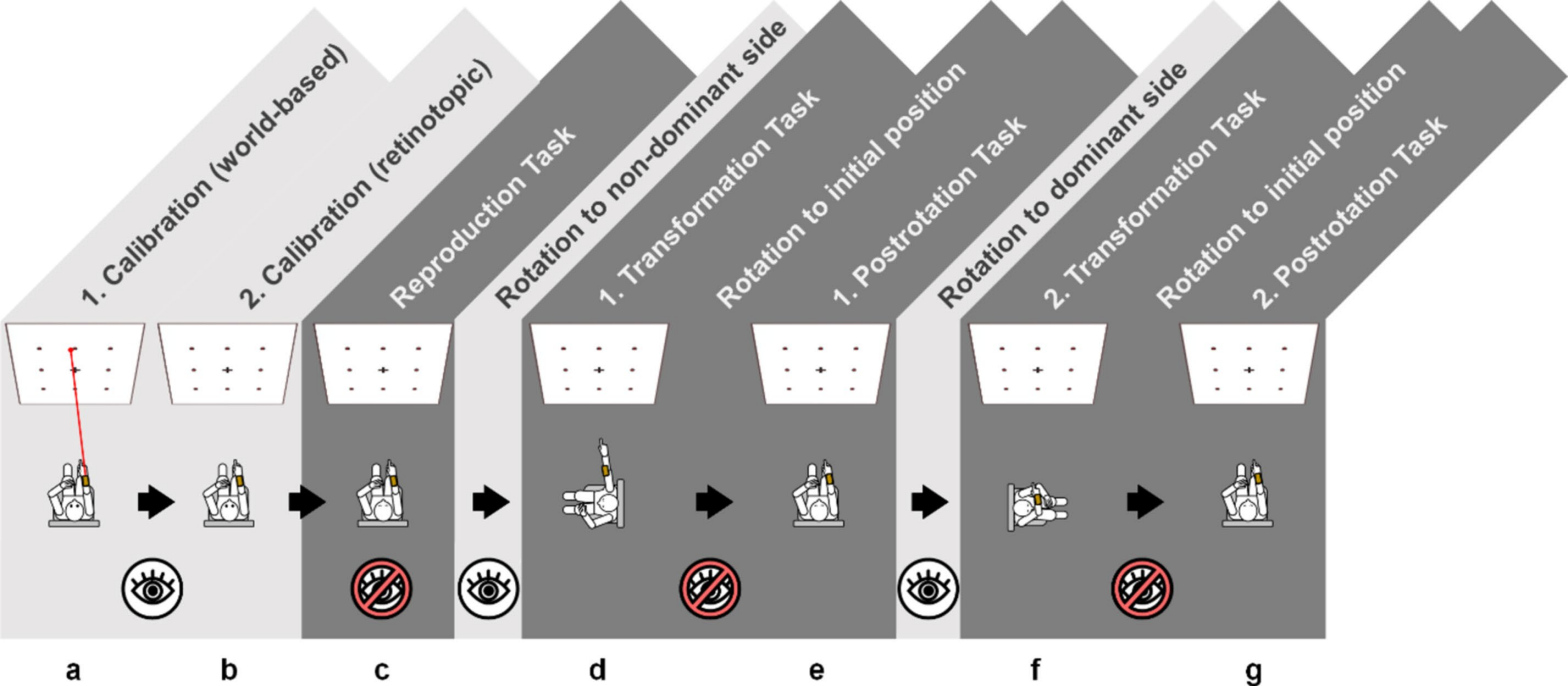
Schematic overview of the 3D-RWPT, with two calibration paradigms (a) world-based calibration with a laser pointer as a visual aid of the real-world pointing vector; (b) retinotopic calibration without a laser pointer, requiring the subject to visually align the target and their finger’s retinotopic representation and five test paradigms (c) initial reproduction with eyes closed in the starting position; (d,f) following a 90° rotation to the side; (e,g) following a 90° rotation back to the starting position. The rotations away from the starting position were performed with visual information available (i.e., eyes open, light grey background) while the rotations towards the initial position and all test paradigms were performed without visual feedback (i.e., eyes closed, dark grey background). Through all paradigms, the targets are given in randomized order, and the pointing vector is recorded with a smartphone-based pointing device. Note that for left-handed participants, the order of rotations would be inverted, meaning all participants were rotated towards their non-dominant side first.

The clinical pointing task was recorded using a smartphone-based pointing device (Flanagin et al., 2019) and the testing setup from previous work (Gerb et al., 2023a; Gerb et al., 2022) and included two calibration and five testing paradigms (Figure 1). Typically, testing (including participant instructions) took 7 to 10 min depending on participant compliance. Patients used their dominant arm (as determined by the EHI) for the test. For the test, participants were seated on a swivel chair with their eye level aligned with the center row of a 3×3 rectangular matrix (angular scope of 55° x 55° in azimuth and polar directions) marked using 20 mm diameter red points on a white wall at a distance of 192 cm. For each task, a computerized voice from the pointing device attached to the participants forearm gave a command, e.g., “top left,” and the subjects pointed towards the target with an extended arm. The volume of the commands was adjustable to ensure sufficient understanding, and instructions were repeated by the test supervisor, if necessary. Each measurement was then confirmed by the examiner using a wireless Bluetooth dongle. Calibration (i.e., pointing to all targets in randomized order with open eyes and visual feedback available) was performed twice: first for the world-based calibration with a laser pointer (visualizing the real-world pointing vector) attached to the device, and then again for the retinotopic calibration, i.e., without the laser pointer, and visual alignment of target and extended index finger. If participants were unable to perform the calibration steps, the experiment was terminated at this stage. After both calibrations (Figures 1a,b), the subjects were asked to point to the targets in newly randomized orders (indicated by the device) without visual feedback while facing straight ahead (Figure 1c), after being passively 90° rotated to their “non- dominant-hand” side (i.e., towards the left side for right-handed participants, towards the right side for left-handed participants, Figure 1d), back in the initial position (Figure 1e), after being passively rotated 90° to their “dominant-hand” side (Figure 1f) and back to the initial target-facing position (Figure 1g). Each test was separated by a standardized pause of 30 s signaled in five-second intervals using a notification sound. Participants who showed a relevant egocentric fallback in the rotation tasks [i.e., pointing as if no rotation had taken place (Gerb et al., 2023a)] were documented by the examiner. Note that the rotations away from the starting position were performed with visual information available (i.e., open eyes, light grey background in Figure 1) while the rotations towards the initial position were performed without visual feedback (i.e., eyes closed, dark grey background in Figure 1).

### 3D-RWPT data analysis

The pointing vectors from each 3D-RWPT paradigm were used to calculate mean angular deviations in the azimuth (≘horizontal) and polar (≘ vertical) planes relative to the two sets of calibrations, as described in previous studies (Gerb et al., 2023a; Gerb et al., 2024a). The mean absolute deviation between each pointing vector and either the world-based or retinotopic calibration vector was computed as a marker of participant performance with lower mean deviations equaling a higher accuracy (lowest possible value: 0°). The deviation was calculated in azimuth (mean azimuth deviation, mAD) and polar (mean polar deviation, mPD) planes for all five tasks individually and grouped for the initial reproduction task, the transformation tasks (when rotated to the dominant and non-dominant side) and the postrotational tasks. The preferred spatial encoding strategy was calculated as previously described (Gerb et al., 2024a). In short, first paradigm-specific angular deviations from either retinotopic or world-based calibration were calculated. Afterwards, the deviation calculated using the world-based calibration was subtracted from the deviation calculated using the retinotopic calibration, resulting in negative values in predominantly retinotopic/egocentric spatial encoding, and positive values in predominantly world-based/allocentric spatial encoding.

### Statistical data analysis

All data was irreversibly anonymized for further analyses and processed using JASP (Version 0.18.3, jasp-stats.org). For data description, we used mean values and standard deviations for continuous variables and absolute and relative frequencies for categorical variables. We tested statistical inference using Spearman’s rho and used an independent samples *t*-test (Welch’s *t*-test) for group comparisons. Additional analyses were performed using ANOVA-testing with Bonferroni-correction for post-hoc comparisons. Correlation analyses and group comparisons were performed for patient age, MoCA scores, mean caloric excitability, state and trait anxiety levels, SBSODS scores (overall score and the three subsets), EISOD scores (overall score and the four subsets), 3D-RWPT performance in azimuth and polar direction and the spatial encoding strategy in azimuth and polar direction. To determine the predictive value of the questionnaires, linear regression models were used.

## Results

135 patients (72 females, 63 males, mean age 62.75 ± 14.46 years) were enrolled in this study. 49 patients showed a relevant cognitive deficit in the MoCA screening test (for demographic data, see Table 1). These had a higher average age compared to the cohort with normal cognition (71.11 ± 10.72 years vs. 57.97 ± 14.41 years; Welch’s *t*-test t − 6.02, *p* < 0.001, Cohen’s d − 1.03), and significantly lower MoCA scores (22.04 ± 2.58 points vs. 28.33 ± 1.43 points; Welch’s *t*-test t 15.73, *p* < 0.001, Cohen’s d 3.01). Both patient groups, with and without cognitive impairment, did not differ in their mean caloric excitability (19.33 ± 11.52°/s vs. 20.53 ± 10.38°/s, Welch’s *t*-test n.s.), or their mean scores in a depression screening test (7.13 ± 4.79 vs. 7.91 ± 5.34, Welch’s *t*-test n.s.)

**Table 1:** Demographic overview of the patient cohorts.

	Normal cognition cohort	(Suspected) cognitive impairment cohort	Group difference (Welch’s *t*-test)
N (of which female)	86 (52)	49 (23)	–
N left-handed	6	3	–
Time course (episodic/chronic)	(34/52)	(8/41)	–
Mean age	57.97 ± 14.41	71.11 ± 10.72	***t*** **− 6.02, *p* < 0.001, Cohen’s d − 1.03**
MoCA	28.33 ± 1.43 (26–30)	22.04 ± 2.58 (15–25)	***t* 15.73, *p* < 0.001, Cohen’s d 3.01**
State anxiety level (in %)	34.27 ± 16.69	36.05 ± 15.06	n.s.
Trait anxiety level (in %)	34.22 ± 14.92	36.32 ± 15.72	n.s.
PHQ-9	7.13 ± 4.79	7.91 ± 5.34	n.s.

Note that “left-handed” refers to the patients’ hand used for the 3D-RWPT, i.e., including ambidextrous patients who chose to perform the 3D-RWPT with their left hand. Time course refers to the suspected main diagnosis; note that no patients with monophasic disease presentation were included. Bold typeset: significant group difference.

Caloric excitability alone did not correlate with EISOD, SBSODS, MoCA scores, patient age, 3D-RWPT-performance or state/trait anxiety levels (Spearman’s rho: n.s.). Female and male patients did not diverge in mean age, MoCA scores, mean caloric excitability, pointing task performance in azimuth and polar direction in the 3D-RWPT or the preferred spatial encoding strategy (Welch’s *t*-test: n.s.).

The patients with polyneuropathy (*n* = 26) did not show relevant group differences in their questionnaire scores (SBSODS, EISOD) when compared to the patients with normal peripheral sensory input (Welch’s *t*-test: n.s.). After correction for patient age, no group differences in their questionnaire scores (SBSODS, EISOD) were observed between chronic and episodic syndromes (ANCOVA with tukey-corrected post-hoc testing: n.s.).

### Effects of state and trait anxiety levels

A first analysis was conducted with state and trait anxiety levels to ensure that both groups (normal cognition and cognitive impairment) exhibited similar levels of general and situational anxiety levels. Here, no group differences were found (Welch’s *t*-test: n.s.). As expected, both state and trait anxiety levels showed no significant correlation with MoCA scores, patient age, or 3D-RWPT angular deviation (Spearman’s rho: n.s.). Both correlated with SBSODS and EISOD scores (Table 2).

**Table 2:** Correlation analysis (Spearman’s rho) of state and trait anxiety levels with SBSODS and SODS scores.

Anxiety level	Spearman’s rho		*p*	Effect size (Fisher’s z)
Trait anxiety				
State anxiety level	**0.56**	***	**< 0.001**	**0.64**
SBSODS	**−0.30**	***	**< 0.001**	**−0.31**
SBSODS_emotional subset	**−0.29**	***	**< 0.001**	**−0.30**
SBSODS_functional subset	**−0.25**	**	**0.003**	**−0.26**
SBSODS_neutral subset	**−0.18**	*	**0.03**	**−0.18**
EISOD_Score	**−0.38**	***	**< 0.001**	**−0.41**
EISOD_Score_MI	**−0.28**	**	**0.001**	**−0.29**
EISOD_Score_MM	**−0.25**	**	**0.004**	**−0.25**
EISOD_Score_N	**−0.33**	***	**< 0.001**	**−0.35**
EISOD_Score_SA	**−0.34**	***	**< 0.001**	**−0.36**
State anxiety				
SBSODS	**−0.29**	***	**< 0.001**	**−0.29**
SBSODS_emotional subset	**−0.33**	***	**< 0.001**	**−0.35**
SBSODS_functional subset	**−0.22**	**	**0.009**	**−0.23**
SBSODS_neutral subset	−0.12	n.s.	0.16	−0.12
EISOD_Score	**−0.47**	***	**< 0.001**	**−0.52**
EISOD_Score_MI	**−0.35**	***	**< 0.001**	**−0.36**
EISOD_Score_MM	**−0.31**	***	**< 0.001**	**−0.32**
EISOD_Score_N	**−0.41**	***	**< 0.001**	**−0.44**
EISOD_Score_SA	**−0.36**	***	**< 0.001**	**−0.38**

Note that almost all subitems of SBSODS and EISOD correlated significantly with both general and situational anxiety levels (bold typeset: significant correlation). *p < 0.05, **p < 0.01, ***p < 0.001.

### Effects of age

Patient age correlated significantly with MoCA scores, the neutral subset of the SBSODS (but not the overall score), the overall EISOD score and most of its subsets except for “scalar abilities” as well as with the mean angular deviation in the overall 3-D-RWPT. Age did not correlate with employed spatial encoding strategy (Table 3).

**Table 3:** Correlation analysis (Spearman’s rho) of patient age with SBSODS and EISOD scores as well as 3D-RWPT performance (mAD = mean absolute azimuth deviation, mPD = mean absolute polar deviation; calculated from retinotopic/egocentric and world-based/allocentric calibration, respectively).

Spearman’s correlations				
	Spearman’s rho		*p*	Effect size (Fisher’s z)
Age				
MoCA	**−0.48**	***	**< 0.001**	**−0.52**
SBSODS	−0.02	n.s.	0.79	−0.02
SBSODS_emotional subset	−0.14	n.s.	0.10	−0.14
SBSODS_functional subset	0.03	n.s.	0.76	0.03
SBSODS_neutral subset	**0.20**	*	**0.02**	**0.21**
EISOD_Score	**−0.25**	**	**0.003**	**−0.26**
EISOD_Score_MI	**−0.19**	*	**0.03**	**−0.19**
EISOD_Score_MM	**−0.29**	***	**< 0.001**	**−0.29**
EISOD_Score_N	**−0.22**	*	**0.01**	**−0.22**
EISOD_Score_SA	−0.11	n.s.	0.21	−0.11
mAD retinotopic	**0.29**	***	**< 0.001**	**0.30**
mAD world-based	**0.31**	***	**< 0.001**	**0.32**
mPD retinotopic	−0.09	n.s.	0.99	−0.09
mPD world-based	**0.18**	*	**0.04**	**0.18**
Azimuth spatial encoding strategy	0.01	n.s.	0.87	0.01
Polar spatial encoding strategy	−0.14	n.s.	0.10	−0.14

Age correlated with MoCA scores, EISOD scores (and all subcategories apart from “scalar abilities”) and the performance in the 3D-RWPT (bold typeset: significant correlation). *p < 0.05, **p < 0.01, ***p < 0.001.

### Effects of cognition

Further correlation analyses were performed with the SBSODS (overall score and subsets), the EISOD (overall score and subsets), and the scores in the cognitive screening test (MoCA). A positive correlation between MoCA scores and SBSODS was primarily driven by the emotional subitems, while the functional and neutral subsets did not correlate with MoCA scores. No reliable association between SBSODS (or subsets) and 3D-RWPT performance was seen; exceptions were the neutral subset and the employed pointing strategy in the polar direction (Spearman’s rho −0.26, p 0.002, Fisher’s z − 0.27), and the overall SBSODS-score (and functional subset) with the azimuth deviation from the world-based/allocentric calibration (Spearman’s rho overall SBSODS -0.18, p 0.04, Fisher’s z − 0.18; functional subset rho −0.17, p 0.05, Fisher’s z − 0.17). An additional analysis of the individual items per subset revealed that only question 5 of the SBSODS neutral subset (“I tend to think of my environment in terms of cardinal directions (N, S, E, W).”) was correlated with the preferred spatial encoding strategy: patients who stated that they thought of their environment in terms of cardinal directions preferred a world-based pointing strategy (Spearman’s rho 0.18, p 0.04, Fisher’s z 0.04).

**Figure 2:**
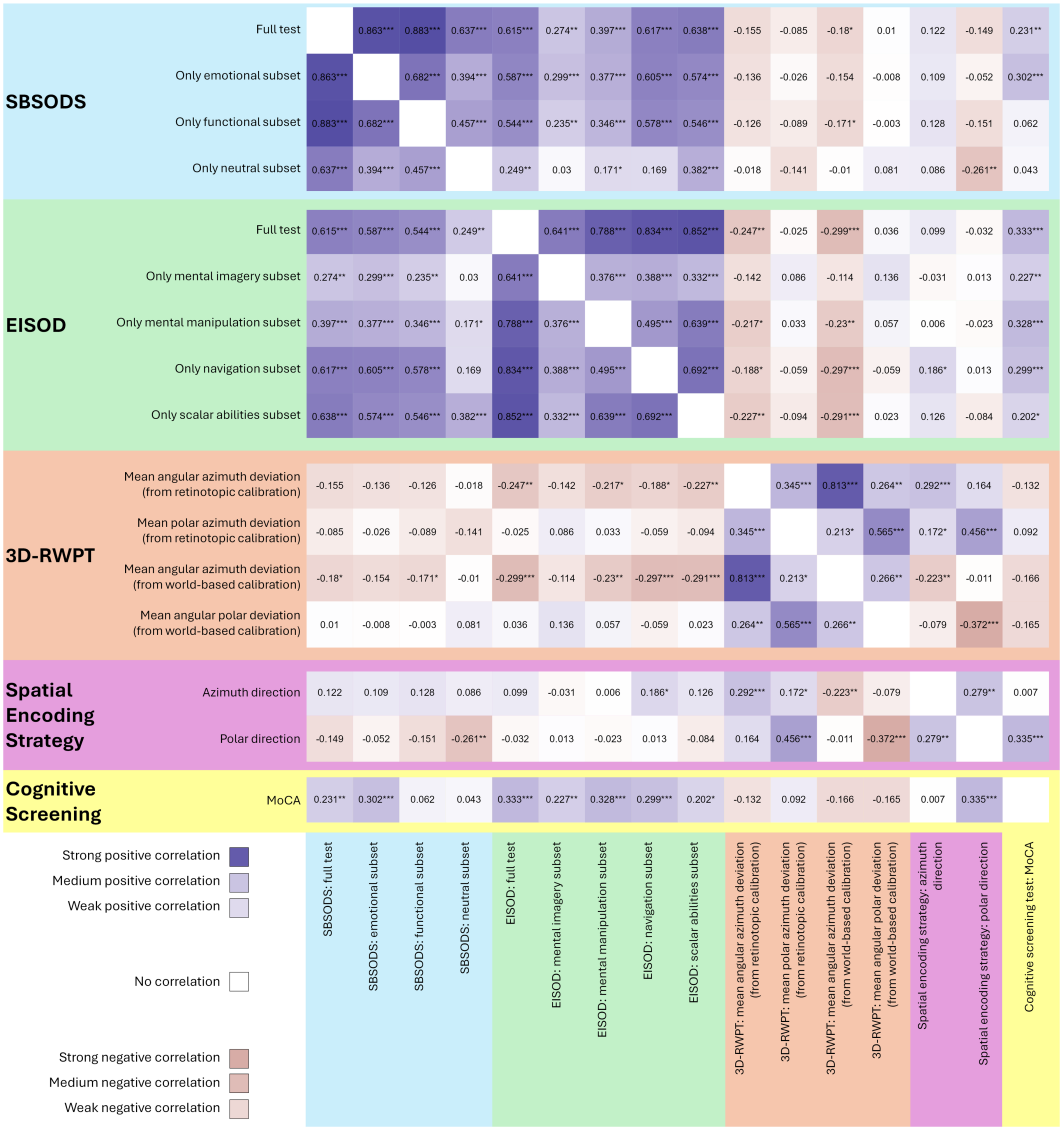
Heatmap of all correlation analyses (Spearman’s rho, positive correlations in blue, negative correlations in pink, stronger colors representing stronger correlation, *p < 0.05, **p < 0.01, ***p < 0.001). Both SBSODS (light blue background) and EISOD (light green background) auto-correlate with their subsets since the overall score is calculated from these subsets. The 3D-RWPT (salmon background) correlates significantly with the EISOD, but only in the azimuth direction. The spatial encoding strategy (purple background) in the polar direction correlates with cognitive function (MoCA scores, yellow background). MoCA scores do correlate with both SBSODS and EISOD scores. However, the correlation with the SBSODS is primarily driven by the subitems with an emotional component, while the functional and neutral subitems show no reliable correlation. For the EISOD, all subcategories correlate with the MoCA scores.

**Figure 3:**
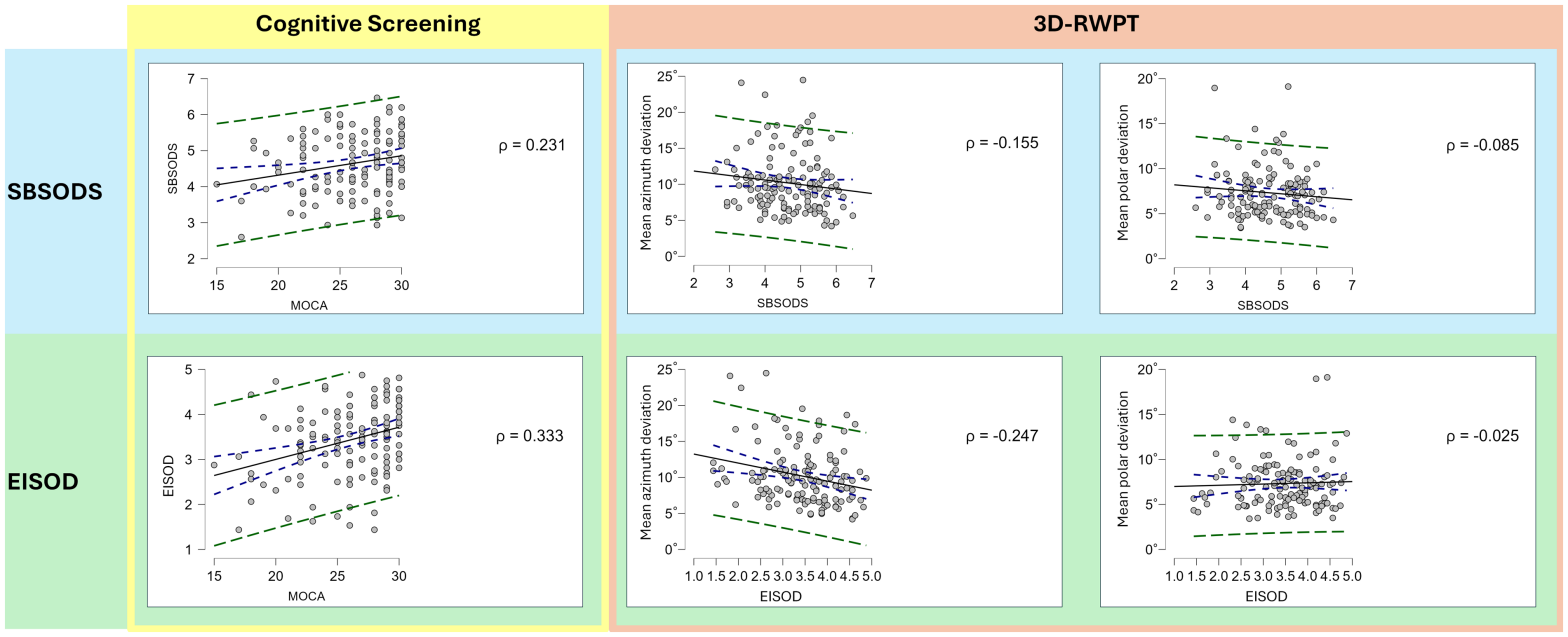
Correlation plots (Spearman’s rho; blue dotted lines: 95% confidence interval; green dotted line: 95% prediction interval). While both lower SBSODS scores (i.e., lower self-reported spatial skills, light blue background) and lower EISOD scores (i.e., higher spatial discomfort levels, light green background) correlate with lower MoCA scores (i.e., cognitive impairment, yellow rectangle), this correlation is primarily driven by the emotional items of the SBSODS (*cf.* Figure 2). Both lower SBSODS scores and lower EISOD scores are associated with worse objective azimuth (but not polar) spatial performance in the 3D-RWPT (salmon rectangle, left side: azimuth deviation, right side: polar deviation; lower deviation equaling better performance), but only the EISOD reaches statistical significance.

The EISOD, however, correlated significantly with the MoCA score, for both the overall score and the subsets (Spearman’s rho overall score 0.33, *p* < 0.001, Fisher’s z 0.35; mental imagery: rho 0.23, p 0.008, z 0.23; mental manipulation: rho 0.33, *p* < 0.001, z 0.34; navigation rho 0.30, *p* < 0.001, z 0.31; scalar abilities rho 0.20, p0.02, z 0.21). Furthermore, the EISOD and all subsets except for “mental imagery” correlated with the (azimuth) angular deviation from both retinotopic/egocentric calibration and world-based/allocentric calibration in the 3D-RWPT (Spearman’s rho overall score −0.25, p0.004, Fisher’s z − 0.25 (retinotopic), rho −0.30, *p* < 0.001, Fisher’s z − 0.31 (world-based); mental manipulation: rho −0.22, p 0.01, z − 0.22 (retinotopic), rho −0.23, p 0.007, Fisher’s z − 0.23 (world-based); navigation rho −0.19, p 0.03, z − 0.19 (retinotopic), rho −0.30, *p* < 0.001, Fisher’s z − 0.31 (world-based); scalar abilities rho −0.23, p 0.008, z − 0.23 (retinotopic), rho −0.29, *p* < 0.001, Fisher’s z − 0.30 (world-based)). Only the navigation subset correlated with the spatial encoding strategy in the azimuth direction (Spearman’s rho 0.19, p 0.03, Fisher’s z 0.19). All correlation analyses are depicted in Figure 2 as a correlation heatmap, while Figure 3 shows selected correlation plots.

On a group level, the cognitively impaired cohort showed significantly lower scores in the emotional subset of the SBSODS (Welch’s *t*-test: t 2.21, p 0.03, Cohen’s d 0.40), but not in the other subsets or the overall SBSODS score (Welch’s *t*-test: n.s.). For the EISOD, overall score and all subtests except for “scalar abilities” showed significant group differences (EISOD: t 2.58, p 0.01, Cohen’s d 0.46; EISOD “mental imagery” t 2.14, p 0.04, Cohen’s d 0.38; EISOD “mental manipulation” t 2.76, p 0.007, Cohen’s d 0.50; EISOD “navigation” t 2.00, p 0.05, Cohen’s d 0.36). To account for multiple comparisons, additional ANOVA-testing was performed (grouped for patient sex and patient cognition, *p*-values adjusted for comparing a family of 4). Again, post-hoc testing confirmed lower scores in the cohort with suspected cognitive impairment (overall SBSODS score: t 2.20, p_Bonf_ 0.03*; SBSODS emotional subset score: t 2.76, p_Bonf_ 6.68×10^−3^; SBSODS functional and neutral subset: n.s.; overall EISOD score: t 2.67, p_Bonf_ 8.54×10^−3^**; EISOD mental manipulation subset: t 2.75, p_Bonf_ 6.73×10^−3^**; EISOD navigation subset: t 2.29, p_Bonf_ 0.02*; EISOD scalar abilities and mental imagery subset: n.s.). Group differences in the 3D-RWPT were only observable in the azimuth plane, narrowly missing statistical significance in the overall deviation (Welch’s *t*-test, retinotopic calibration: t − 1.86, p 0.07; world-based calibration: t − 1.87, p 0.06), but reaching statistical significance in the transformation paradigm subanalysis (Welch’s *t*-test, retinotopic calibration: t − 2.37, p 0.02, Cohen’s d − 0.44; world-based calibration: t − 2.22, p 0.03*, Cohen’s d − 0.41).

**Figure 4:**
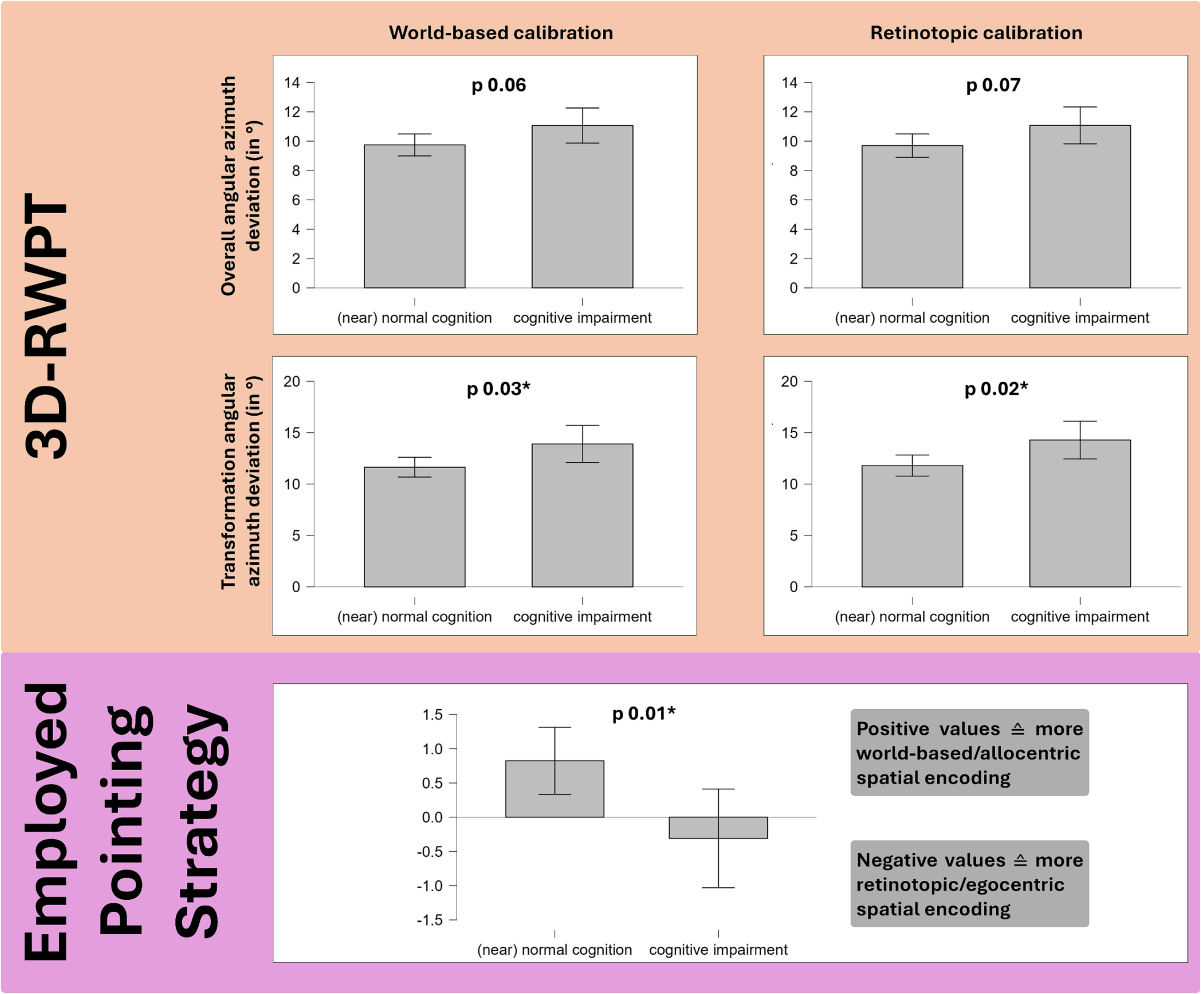
Group comparison (Welch’s *t*-test) of 49 patients with cognitive impairment in the MoCA dementia screening test and 86 patients with normal cognition. Salmon background: the cognitively impaired group showed higher angular deviation in the 3D-RWPT, reaching statistical significance in the (cognitively most demanding) transformation paradigm (cp. Figures 1d,f). Purple background: patients with cognitive impairment used more retinotopic/egocentric spatial encoding strategies (depicted: spatial encoding strategy in the polar plane).

Cognition correlated with the spatial encoding strategy: patients with lower scores in the dementia screening test exhibited more retinotopic spatial encoding. However, this difference was only observable in the polar plane (Spearman’s rho MoCA/polar spatial encoding strategy: 0.34***, *p* < 0.01, Fisher’s z 0.35). On a group level, this effect was also highly significant (Welch’s *t*-test: t 2.61, p 0.01, Cohen’s d 0.47) (see Figure 4).

### Effects of sex

In the psychometric tests, female and male patients showed comparable baseline state and trait anxiety levels (Welch’s *t*-test: n.s.), but male patients reported higher SBSODS scores, i.e., higher subjective spatial abilities, and higher EISOD-scores, i.e., lower spatial discomfort levels (SBSODS: 4.90 ± 0.75 vs. 4.42 ± 0.87, Welch’s *t*-test: t − 3.41, *p* < 0.001; EISOD: 3.65 ± 0.62 vs. 3.24 ± 0.89, Welch’s *t*-test: t − 3.14, p 0.002). In the subset analyses, two notable exceptions were observable: male and female patients did not show significant group differences in the neutral subset of the SBSODS, and in the mental imagery subcategory of the EISOD (Welch’s *t*-test: n.s.).

### Predictive value analysis

In order to determine the predictive value of both questionnaires, linear regression models were used. In the first analysis, the predictive value of EISOD-score and SBSODS-score for the MoCA-score was assessed. Given the expected sex differences, patient sex was used as a factor, and all covariates were entered simultaneously in the model (forced entry). Here, only patient sex and EISOD-score reached statistical significance (EISOD: t 2.84, p 5.15×10^−3^; male sex: t-2.71, p 7.56×10^−3^). However, this model was not able to reliably predict MoCA-scores (R^2^ 0.15, *p* < 0.01). In the second model, the predictive value of the questionnaires factored over patient sex for the mean angular deviation in the transformation paradigm of the 3D-RWPT was analyzed. While this resulted in a very poor fit (R^2^ 0.08, *p* < 0.01 for retinotopic deviation, R^2^ 0.09, p 4.66×10^−3^ for world-based deviation), again only the EISOD-score reached statistical significance as a single coefficient (t − 2.83, p 5.42×10^−3^ for retinotopic deviation, t − 2.92, p 4.15×10^−3^ for world-based deviation).

## Discussion

In the current study of self-assessed questionnaires on spatial anxiety and spatial orientation discomfort compared with the objective performance of a 3D real-world pointing task, patients without and with cognitive impairment were examined. Importantly, all patients underwent detailed neurotological testing to rule out vestibular dysfunction as a potential confounder. The focus was on two questions: first, are the subjective questionnaires reliably associated with deficits in (a) objective orientation performance and (b) a dementia screening test in vestibular healthy patients, second, can the newly introduced extended questionnaire of subjective spatial discomfort (EISOD) provide additional insight into spatial orientation impairment when combined with a questionnaire of subjective spatial abilities (SBSODS)? In the current study, both questionnaires correlated with objective spatial performance, however, the EISOD showed higher correlation indices. When compared to the cognitive screening test, the SBSODS only partially (in its emotional subset) correlated with MoCA scores, while, again, the EISOD showed a stronger correlation. The patient group with suspected cognitive impairment preferred to use retinotopic/ egocentric spatial encoding strategies in the pointing task, while exhibiting higher angular deviations in the accuracy analysis. Furthermore, the latter reported lower subjective spatial skill levels and higher spatial discomfort levels (i.e., lower scores in the EISOD).

Our findings are in line with previous research, which had shown spatial anxiety/discomfort to be a common finding in cognitive impairment and early AD (Cerman et al., 2018). This could potentially be explained by patient’s awareness of their decreasing spatial abilities, or a (understandable) fear of, e.g., getting lost in new places, but might also stem from yet undisclosed vestibular dysfunction. In the study by Cerman et al. (2018), the authors corrected for patient anxiety levels and depressive symptoms, but did not provide vestibular testing results. Importantly, the overlap with vestibular disorders needs to be addressed, since elevated spatial anxiety / discomfort levels were also observed in BVP (Kremmyda et al., 2016) and other peripheral-vestibular disorders (Jáuregui-Renaud et al., 2024), and spatial anxiety / discomfort alone is therefore not necessarily a sign of cognitively modulated visuospatial impairment. Other studies on spatial discomfort in dementia usually neglect vestibular diagnostics (Cerman et al., 2018; Davis and Veltkamp, 2020). In our study, we therefore ensured normal peripheral and central vestibular function in all patients through a detailed, state-of-the-art neurotological assessment. This allowed for a focused analysis of the relationship between (suspected) cognitive impairment and spatial orientation discomfort using the newly introduced EISOD as well as with a second, well-established spatial self-report (SBSODS), without potential vestibular confounders. Furthermore, this approach ensured that (still hardly known) indirect effects of vestibular dysfunction, e.g., due to changes in emotion processing, cannot interfere with the questionnaire scores.

We observed a significant correlation between patient age and questionnaire scores as well as between patient age and 3D-RWPT. Based on the data presented in the current study, no clear analysis of the influence of physiological ageing (vs. effects of pathological cognitive decline, which typically also manifests in older age) was possible. Here, future studies in a large cohort of subjects with normal vestibular function and normal cognition are still required.

As expected, sex differences in self-assessed spatial abilities were observable. This is in line with multiple previous studies of the SBSODS, e.g., by Schinazi et al. (2023), who found participant motivation to be a relevant modulator of these sex differences. Other explanations proposed were internalized belief systems (Crawford et al., 1989) of higher spatial abilities in men directly influencing actual spatial performance (Moè and Pazzaglia, 2010), or different spatial encoding strategies in navigation tasks with male participants preferring global reference points, independent of cultural background (Lawton and Kallai, 2002).

Notably, the SBSODS and the EISOD are test instruments for different aspects of spatial perception: the SBSODS reports the participant’s subjective skill level in various spatial domains, whereas the EISOD depicts the subject’s discomfort in spatial tasks. There is no mandatory dependency of the skill level on the degree of discomfort: subjects with high (subjective or objective) skill levels in orientation and navigation tasks can still feel discomfort or stress in these situations. Here, a complimentary usage of both SBSODS and EISOD (or similar test instruments) seems promising, since both aspects of spatial impairment can considerably contribute to the differential diagnosis (Cerman et al., 2018).

In the pointing task (3D-RWPT), findings from our previous studies (Gerb et al., 2024a; Gerb et al., 2023a) could be confirmed: patients with suspected cognitive impairment struggled most in the body rotation transformation paradigms. These paradigms require a corresponding internal mental rotation of the calibration reference frame to the changed body orientation in space, to adjust their pointing movements accordingly. Such an update of the internal representation of the environment appears to be most sensitive to a cognitive decline. Furthermore, the relative stability of pointing performance in the polar plane compared to the azimuth plane corresponds to an earlier observation (Gerb et al., 2023a). While azimuth angular deviation increased with higher age, polar angular deviations were generally lower and remained stable over the entire age range. The reasons for this may reflect a basic anisotropy of human horizontal versus vertical spatial encoding (Brandt et al., 2015; Zwergal et al., 2016) with human locomotion being predominantly ground-based, i.e., horizontal. This is particularly relevant for younger individuals with more locomotor activity and applies not only to humans but also to all ground-based species, e.g., rodents (Jovalekic et al., 2011). Alternatively, a potential reason could be the test design of the 3D-RWPT, which only involves a yaw-axis rotation, but no pitch-axis changes. Here, future experiments could include rotation or translation in other planes (e.g., around the pitch-axis), which potentially result in more pronounced polar (vertical) angular deviations. This aspect would be particularly interesting in BVP patients with remaining otolith function (i.e., normal saccular and utricular testing results), or patients with isolated otolith dysfunction.

## Conclusions and limitations

In general, the application of the extended questionnaire (EISOD) largely correlated with the test results of objective spatial skills in the pointing task (3D-RWPT). Both tests, i.e., subjective spatial discomfort and subjective spatial abilities, as well as the objective spatial test, correlated to some extent with the degree of cognitive impairment determined by a dementia screening test. The newly compiled EISOD (assessing subjective spatial discomfort) showed significantly higher concordance with cognitive screening tests and objective spatial performance than the SBSODS, which assesses subjective spatial abilities.

A potential limitation of this primarily methodical study is the patient heterogeneity. The cohort of cognitively impaired patients was not further classified as to their various etiology, pathophysiology, and course of disease. This requires future studies on the differential performance of various forms of dementia, because subjective and objective test results may diverge. Furthermore, it should be noted that we intentionally excluded patients with acute or chronic peripheral or central vestibular deficits in the present study, because, e.g., a bilateral vestibular loss causes significant spatial memory and navigational deficits, as could be shown in rodents (Smith, 2023) and humans (Schöberl et al., 2021; Gammeri et al., 2022; Dobbels et al., 2019). Further research in patients with additional vestibular dysfunction is needed to understand the impact of disturbances in the vestibular system on spatial abilities in general, and the emotional processing of spatial impairment in particular. As shown earlier, cognitive and vestibular deficits caused different patterns of spatial impairment in the 3D-RWPT which were particularly pronounced when both conditions occurred simultaneously (Gerb et al., 2024a). Moreover, it has been shown in an MRI meta-analysis that vestibular and anxiety networks overlap (Neumann et al., 2023) and that vestibular function affects anxiety (Dieterich and Brandt, 2024). With respect to clinical application of subjective questionnaires and objective measures, we recommend using multiple methods in parallel, at least as bedside screening procedures. In patients with normal vestibular function, higher spatial anxiety / discomfort levels can be assumed to be related to cognitive impairment, as shown in the current study. Further studies are required to elucidate the relationship between physiological aging, vestibular dysfunction, and spatial orientation discomfort.

## Data Availability

The raw data supporting the conclusions of this article will be made available by the authors, without undue reservation.
