# Supplementary material for: Subjective spatial orientation discomfort is associated with decreased real-world spatial performance and lower cognitive function
Source: Front Neurosci. 2024 Nov 13;18:1481653. doi: 10.3389/fnins.2024.1481653 (PMC11599218; doi:10.3389/fnins.2024.1481653)
Supplement: Supplementary file 1 [file Table_1.DOCX]

# Suppl. Data for Gerb et al: Self-assessment of spatial orientation discomfort, a useful predictor of 3D real world spatial test results and cognitive function

For the subscores, calculate the average response (very anxious/highly stressed: 1 point; not anxious/discomfortable at all: 5 points) for the following items:

Mental Imagery: items 1, 2, 3, 4

Mental Manipulation: items 5,6,10,16

Navigation: items 7, 11, 12, 13

Scalar abilities: items 8, 9, 14, 15

Note that the individual items stem from the following publications:

Items 1, 2, 3, 5, 6, 7, 8, 9, 10, 16

Lyons, I. M., Ramirez, G., Maloney, E. A., Rendina, D. N., Levine, S. C., & Beilock, S. L. (2018). Spatial Anxiety: A Novel Questionnaire With Subscales for Measuring Three Aspects of Spatial Anxiety. *Journal of Numerical Cognition*, *4*(3), 526-553. <https://doi.org/10.5964/jnc.v4i3.154>

Items 4, 14, 15

Geer, E. A. (2019). The Development and Construct Validation of the Revised Spatial Anxiety Scale. Retrieved from <http://purl.flvc.org/fsu/fd/2019_Fall_Geer_fsu_0071N_15592>

Items 11, 12

Lawton, C.A. Gender differences in way-finding strategies: Relationship to spatial ability and spatial anxiety. *Sex Roles* **30**, 765–779 (1994). <https://doi.org/10.1007/BF01544230>

Item 13

Malanchini M, Rimfeld K, Shakeshaft NG, Rodic M, Schofield K, Selzam S, Dale PS, Petrill SA, Kovas Y. The genetic and environmental aetiology of spatial, mathematics and general anxiety. Sci Rep. 2017 Feb 21;7:42218. doi: 10.1038/srep42218

Please ensure to reference these works when using the EISOD, e.g., “Participants filled out the Extended Inventory for Spatial Orientation Discomfort (EISOD, Gerb et al. (2024)), which utilizes items from previously validated scales for spatial anxiety and discomfort (Lyons et al. (2018), Geer (2019), Lawton (1994), Malanchini (2017)).”

Extended Inventory of Spatial Orientation Discomfort (EISOD)

| **EISOD** | | | | | |
| --- | --- | --- | --- | --- | --- |
| Please indicate how anxious, stressed or discomfortable the following tasks (e.g., in an exam) would make you feel, and tick one of the answer options. | | | | | |
|  | Very anxious/ highly discomfortable | Quite anxious / quite discomfortable | Somewhat anxious/discomfortable | Only a little anxious/discomfortable | Not anxious/discomfortable at all |
| Being asked to draw a detailed picture of a key landmark in your neighbourhood | □ | □ | □ | □ | □ |
| Being asked to recall the shade and pattern of a person's tie  you met for the first time the previous evening | □ | □ | □ | □ | □ |
| Being asked to draw the front of an imaginary building. | □ | □ | □ | □ | □ |
| Being asked to locate a specific object among a scene with  many random objects in it. | □ | □ | □ | □ | □ |
| Being asked to imagine and mentally rotate a 3-dimensional  figure | □ | □ | □ | □ | □ |
| Being asked to write the alphabet with the letters upside-down  as quickly and accurately as possible. | □ | □ | □ | □ | □ |
| Trying to get somewhere you have never been to before  in the middle of an unfamiliar city | □ | □ | □ | □ | □ |
| Asked to find a location on a topographical map using  information from the contour lines. | □ | □ | □ | □ | □ |
| Tested on your ability to estimate the shortest route by  air between two cities on a globe | □ | □ | □ | □ | □ |
| Tested on your ability to solve a complicated 3-  dimensional puzzle | □ | □ | □ | □ | □ |
| Leaving a store that you have been to for the first time  and deciding which way to turn to get to a destination. | □ | □ | □ | □ | □ |
| Finding your way out of a complex arrangement of  offices that you have visited for the first time. | □ | □ | □ | □ | □ |
| Finding your way around an intricate arrangement of  streets. | □ | □ | □ | □ | □ |
| Measuring the distance between two cities on a map  using the scale provided. | □ | □ | □ | □ | □ |
| Using blueprints of a building to determine the location  of an object in one of the rooms. | □ | □ | □ | □ | □ |
| Imagining on a test what a 3-dimensional landscape  model would look like from a different point of view | □ | □ | □ | □ | □ |

German Version of the Extended Inventory of Spatial Orientation Discomfort (EISOD)

| **EISOD** | | | | | |
| --- | --- | --- | --- | --- | --- |
| Bitte überlegen Sie, als wie unangenehm oder stressig Sie die folgenden Aufgaben (zum Beispiel in einer Prüfung) empfinden würden, und kreuzen Sie jeweils eine der Antwortmöglichkeiten an. | | | | | |
|  | Sehr unangenehm | Ziemlich unangenehm | Eher unangenehm | Ein wenig unangenehm | Gar nicht unangenehm |
| Ein detailliertes Bild einer Sehenswürdigkeit aus Ihrer Nachbarschaft malen zu müssen. | □ | □ | □ | □ | □ |
| Sich an die genaue Farbe und das Muster der Krawatte einer Person erinnern zu müssen, die Sie am Abend zuvor zum ersten Mal gesehen haben. | □ | □ | □ | □ | □ |
| Sich die Fassade eines Hauses ausdenken und diese malen zu müssen. | □ | □ | □ | □ | □ |
| In einem unübersichtlichen Bild ein bestimmtes Objekt finden zu müssen. | □ | □ | □ | □ | □ |
| Sich eine dreidimensionale Figur vorstellen und diese im Kopf drehen zu müssen. | □ | □ | □ | □ | □ |
| Das Alphabet möglichst schnell und genau so schreiben zu müssen, dass alle Buchstaben auf dem Kopf stehen. | □ | □ | □ | □ | □ |
| Zu einem Ihnen unbekannten Ort in einer fremden Stadt gelangen zu müssen. | □ | □ | □ | □ | □ |
| Nur anhand der Höhenlinien einer Landkarte einen bestimmten Ort finden zu müssen. | □ | □ | □ | □ | □ |
| Den Abstand (Luftlinie) zwischen zwei Städten schätzen zu müssen. | □ | □ | □ | □ | □ |
| Ein kompliziertes, dreidimensionales Puzzle lösen zu müssen. | □ | □ | □ | □ | □ |
| Entscheiden zu müssen, in welche Richtung Sie weitergehen müssen, um an Ihr nächstes Ziel zu gelangen, wenn Sie aus einem Ihnen unbekannten Geschäft herauskommen. | □ | □ | □ | □ | □ |
| Den Weg aus einem Ihnen unbekannten Bürogebäude finden zu müssen, in dem Sie heute zum ersten Mal waren. | □ | □ | □ | □ | □ |
| Ihren Weg durch viele verwinkelte kleine Straßen finden zu müssen. | □ | □ | □ | □ | □ |
| Auf einer Landkarte den Abstand zwischen zwei Städten ausmessen zu müssen. | □ | □ | □ | □ | □ |
| Anhand des Bauplans eines Gebäudes ein Objekt in einem der Räume finden zu müssen. | □ | □ | □ | □ | □ |
| Sich vorstellen zu müssen, wie ein dreidimensionales Modell einer Landschaft aus einem anderen Blickwinkel aussieht. | □ | □ | □ | □ | □ |
